# Preclinical evaluation of new GRPR-antagonists with improved metabolic stability for radiotheranostic use in oncology

**DOI:** 10.1186/s41181-024-00242-6

**Published:** 2024-02-16

**Authors:** Panagiotis Kanellopoulos, Adam Mattsson, Ayman Abouzayed, Karim Obeid, Berthold A. Nock, Vladimir Tolmachev, Theodosia Maina, Anna Orlova

**Affiliations:** 1 Department of Medicinal Chemistry, Uppsala University, 75183 Uppsala, Sweden; 2 Molecular Radiopharmacy, INRaSTES, NCSR “Demokritos”, 15341 Athens, Greece; 3 Department of Immunology, Genetics and Pathology, Uppsala University, 75183 Uppsala, Sweden; 4 Science for Life Laboratory, Uppsala University, 75237 Uppsala, Sweden

**Keywords:** Prostate cancer, GRPR-antagonist, Radiotheranostics, PC-3 tumors, Metabolic stability, NEP-inhibition

## Abstract

**Background:**

The gastrin-releasing peptide receptor (GRPR) has been extensively studied as a biomolecular target for peptide-based radiotheranostics. However, the lack of metabolic stability and the rapid clearance of peptide radioligands, including radiolabeled GRPR-antagonists, often impede clinical application. Aiming at circumventing these drawbacks, we have designed three new GRPR-antagonist radioligands using [^99m^Tc]Tc-DB15 ([^99m^Tc]Tc-N_4_-AMA-DIG-_D_Phe-Gln-Trp-Ala-Val-Sar-His-Leu-NHEt; AMA: *p*-aminomethylaniline; DIG: diglycolate) as a motif, due to its high GRPR-affinity and stability to neprilysin (NEP). The new analogues carry the DOTAGA-chelator (1,4,7,10-tetraazacyclododecane-1-glutaric acid-4,7,10-triacetic acid) through different linkers at the N-terminus to allow for labeling with the theranostic radionuclide pair In-111/Lu-177. After labeling with In-111 the following radioligands were evaluated: (i) [^111^In]In-AU-SAR-M1 ([^111^In]In-DOTAGA-AMA-DIG-_D_Phe-Gln-Trp-Ala-Val-Sar-His-Leu-NHEt), (ii) [^111^In]In-AU-SAR-M2 ([^111^In]In-[DOTAGA-Arg]AU-SAR-M1) and (iii) [^111^In]In-AU-SAR-M3 ([^111^In]In-[DOTAGA-_D_Arg]AU-SAR-M1).

**Results:**

These radioligands were compared in a series of in vitro assays using prostate adenocarcinoma PC-3 cells and in murine models. They all displayed high and GRPR-specific uptake in PC-3 cells. Analysis of mice blood collected 5 min post-injection (pi) revealed similar or even higher metabolic stability of the new radioligands compared with [^99m^Tc]Tc-DB15. The stability could be further increased when the mice were treated with Entresto® to in situ induce NEP-inhibition. In PC-3 xenograft-bearing mice, [^111^In]In-AU-SAR-M1 displayed the most favourable biodistribution profile, combining a good tumor retention with the highest tumor-to-organ ratios, with the kidneys as the dose-limiting organ.

**Conclusions:**

These findings strongly point at AU-SAR-M1 as a promising radiotherapeutic candidate when labeled with Lu-177, or other medically appealing therapeutic radiometals, especially when combined with in situ NEP-inhibition. To this goal further investigations are currently pursued.

**Supplementary Information:**

The online version contains supplementary material available at 10.1186/s41181-024-00242-6.

## Background

Prostate cancer (PC) is the fourth most commonly diagnosed cancer worldwide and the second most common in men (Sung et al. 2021). PC incidence rates are notably higher in high development index countries, and are rapidly escalating in regions with increasing life expectancy too (Sung et al. 2021). There are currently three main tools for diagnosis of prostate cancer, prostate specific antigen (PSA) blood measurement, digital rectal examination and Gleason scoring of biopsy material. Gleason scoring is also useful for cancer staging and therapy planning (Faviana et al. 2021). Patient management and treatment options heavily rely on accurate detection, staging and spread assessment of the disease. Thus, diagnostic tools are needed which can provide results accurately, fast and non-invasively, thereby minimizing biopsy-associated human error and patient discomfort. An emerging field for diagnostic and therapeutic treatment of cancer is radiotheranostics, employing target-specific radionuclide carriers for diagnostic imaging with positron emission tomography (PET) (e.g. F-18, Ga-68, Zr-89) or single photon emission computed tomography (SPECT) (e.g. Tc-99m, In-111) often combined with computed tomography (CT), and for delivering radiotoxic doses to eradicate tumor lesions (e.g. I-131, Lu-177, Ac-225) (Dumont et al. 2013).

The gastrin-releasing peptide receptor (GRPR) represents a promising target in prostate cancer radiotheranostics. It is a member of the bombesin family of receptors and it is overexpressed in 63–100% of all prostate cancer cases while having a low expression level in healthy tissues of the body, with the exception of the pancreas and the gastrointestinal tract (Beer et al. 2012; Mansi et al. 2021; Patel et al. 2006; Rinne et al. 2021). It has been found that GRPR expression more clearly correlates to Gleason scoring than prostate specific membrane antigen (PSMA), with higher expression evident in early-stage prostate cancer and, most importantly, minimal expression in prostate hyperplasia (Faviana et al. 2021). Thus, diagnostic imaging of GRPR expression might be a valuable tool in the early discovery of prostate cancer, confined or already infiltrating in the surrounding tissues, providing more therapeutic options for patients.

Bombesin (BBN, Pyr-Gln-Arg-Leu-Gly-Asn-Gln-Trp-Ala-Val-Gly-His-Leu-Met-NH_2_), first isolated from the European fire-bellied toad *Bombina bombina* in 1971, is known for its high affinity for the GRPR (Anastasi et al. 1971). Bombesin analogues, particularly C-terminal peptide fragments, have extensively been investigated as templates for the development of radiotheranostics (Ferreira et al. 2017; Mansi et al. 2021; Moreno et al. 2016). Early efforts were focused on receptor agonists, because rapid internalization into tumor cells after receptor binding of such radioligands was believed to be crucial for high and sustained tumor uptake (Mansi et al. 2021). In practice, GRPR activation by agonists triggered acute adverse effects mainly in the gastrointestinal system and was also associated with mitogenic actions (Bodei et al. 2007; Rozengurt et al. 1990). Therefore, a shift in GRPR-radioligand development towards antagonists occurred following the trends in neuroendocrine tumor radiotheranostics based on somatostatin (Haider et al. 2020). Several studies have demonstrated that radiolabeled GRPR antagonists achieve higher tumor uptake and retention in comparison with agonists, despite their significantly inferior internalization in tumor cells (Cescato et al. 2008; Maina et al. 2017; Mansi et al. 2009; Mitran et al. 2020; Nock et al. 2005). Furthermore, their short amino acid chains, in combination with the structural changes introduced in the native sequence lead to enhanced resistance to degrading peptidases in the blood (Schottelius and Wester 2009).

**Fig. 1:**
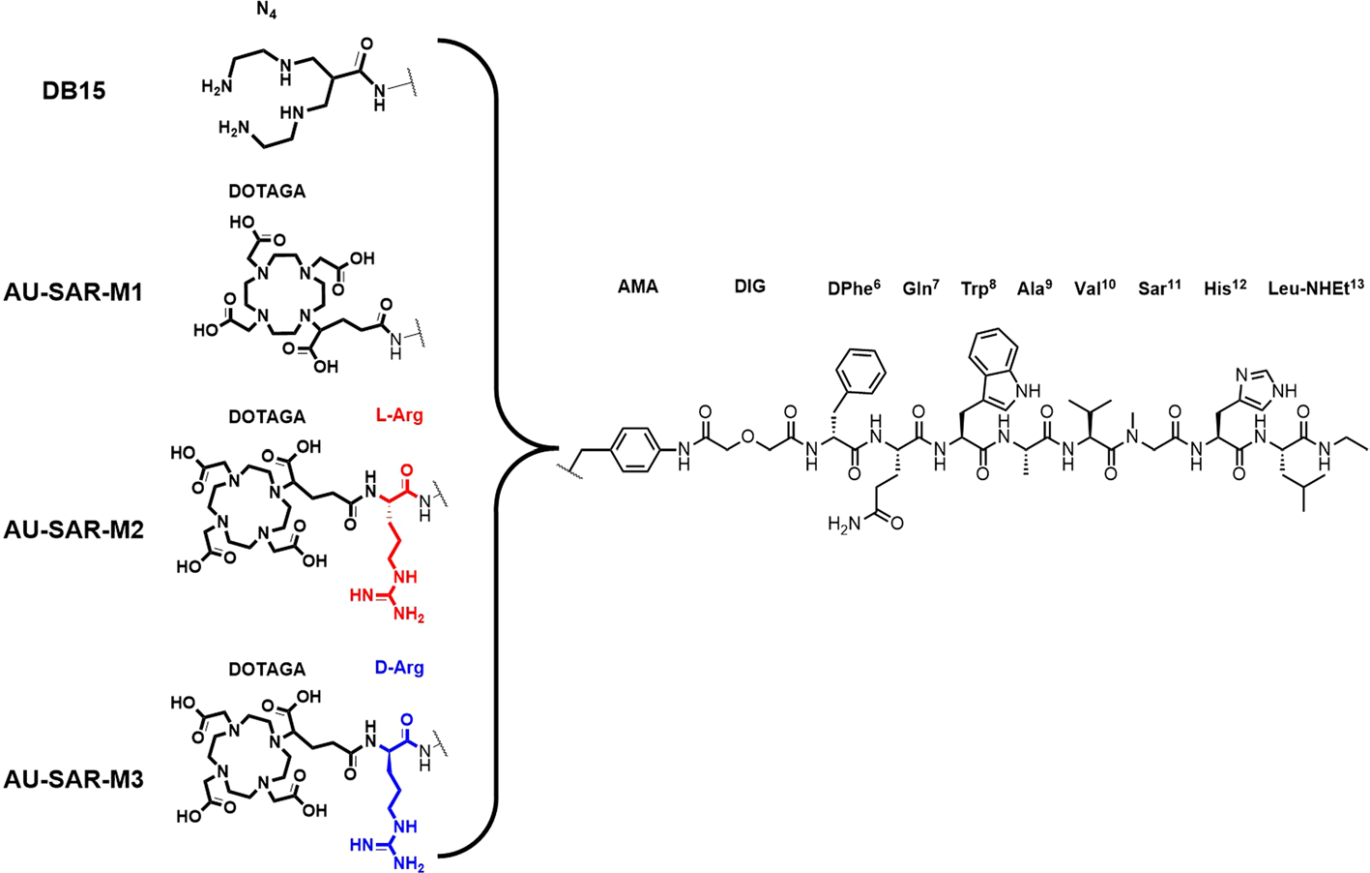
Chemical structures of DB15, AU-SAR-M1, AU-SAR-M2 and AU-SAR-M3

A very promising motif in the design of GRPR-antagonist based radiopharmaceuticals is [_D_Phe^6^,Leu^13^-NHEt]BBN(6–13) (Wang et al. 1990a; b), exemplified by Demobesin 1 and its mimics (DB1, N_4_′-DIG-[_D_Phe^6^,Leu-NHEt^13^]BBN(6–13); N_4_′: 6-{*p*-[(carboxymethoxy)acetyl]-aminobenzyl}-1,4,8,11-tetraazaundecane; DIG, diglycolate) for labeling with Tc-99m (Nock et al. 2003, 2018), or SB3 (DOTA-AMA-DIG-[_D_Phe^6^,Leu-NHEt^13^]BBN(6–13); DOTA = 1,4,7,10-tetraazacyclododecane-1,4,7,10-tetraacetic acid; AMA, *p*-aminomethylaniline) for labeling with trivalent radiometals (Bakker et al. 2021; Lymperis et al. 2018; Maina et al. 2016). The resulting radioligands demonstrated good GRPR-affinity and cell uptake, high tumor accumulation and rapid clearance from the background in mice, even from the GRPR-rich pancreas (Mansi et al. 2021; Nock et al. 2003). However, their stability in the circulation was found to be compromised by the proteolytic action of neprilysin (NEP) (Lymperis et al. 2018). Interestingly, the Gly^11^/Sar^11^-modified radioligand [^99m^Tc]Tc-DB15 (Fig. 1. N_4_-AMA-DIG-[_D_Phe^6^,Sar^11^,LeuNHEt^13^]BBN(6–13); N_4_, 6-carboxy-1,4,8,11-tetraazaundecane)) (Nock et al. 2021, 2018), turned out to be NEP-resistant, showing improved GRPR-mediated tumor uptake and retention in murine models. Most importantly, [^99m^Tc]Tc-DB15 demonstrated excellent tolerability and good lesion identification efficacy in a pilot study performed in a small number of advanced breast cancer patients (Nock et al. 2021).

Recent studies have revealed the potential of radiolabeled GRPR antagonists for therapeutic applications in human cancer. Thus, the antagonists RM2 (DOTA-4-amino-1-carboxymethyl-piperidine-_D_Phe-Gln-Trp-Ala-Val-Gly-His-Sta-Leu-NH_2_) and DOTAGA-PEG_2_-RM26 (RM26, [_D_Phe^6^,Sta^13^,Leu^14^-NH_2_]BBN(6–14)) labeled with Lu-177 displayed favourable biodistribution profiles and managed to extend survival in murine prostate cancer models (Dumont et al. 2013; Mitran et al. 2019). A first-in-human study showed that the administration of [^177^Lu]Lu-RM2 antagonist was well tolerated in patients with the GRPR-rich pancreas being the dose-limiting organ (Kurth et al. 2020). In order to enhance therapeutic efficacy, the search for GRPR-radioantagonists with improved metabolic stability and prolonged retention times on tumor-lesions is currently ongoing (D’Onofrio et al. 2023).

In view of the above, in the present work we aimed to develop an improved radiotheranostic agent using the NEP-resistant [^99m^Tc]Tc-DB15 as a template. Hence, we designed three new DB15 analogues coupled to DOTAGA (1,4,7,10-tetraazacyclododecane-1-glutaric acid-4,7,10-triacetic acid) via three different linkers. Unlike the open-chain tetraamine in DB15, suitable for labeling with the SPECT radionuclide Tc-99m, DOTAGA allows for labeling with a wider panel of trivalent radiometals of medical relevance, offering better perspectives for radiotheranostic applications. Thus, first the effect of chelator-switch from N_4_ to DOTAGA was studied in AU-SAR-M1 (DOTAGA-AMA-DIG-[_D_Phe^6^,Sar^11^,NHEt^13^]BBN(6–13)). Secondly, the introduction of a basic _L/D_-Arg next to DOTAGA was pursued in order to compensate for the positive charge loss of the N-terminal monocationic [[^99m^Tc]Tc(O)_2_(N_4_)]^+1^ metal-chelate, since a positive charge at the N-terminus of bombesin analogues has been associated with high receptor affinity (Abouzayed et al. 2020). The latter linker modifications afforded AU-SAR-M2 (DOTAGA-Arg-AMA-DIG-[_D_Phe^6^,Sar^11^,NHEt^13^]BBN(6–13)) and AU-SAR-M3 (DOTAGA-_D_Arg-AMA-DIG-[_D_Phe^6^,Sar^11^,NHEt^13^]BBN(6–13)) (Fig. 1). The new DOTAGA-bioconjugates were labeled with the broadly available trivalent radiometal In-111 and their properties were compared in vitro (GRPR affinity, specificity and uptake in prostate adenocarcinoma PC-3 cells), as well as in vivo (metabolic stability in peripheral blood of healthy mice, biodistribution in PC-3 xenograft-bearing mice) to reveal the compound of choice for further study as a Lu-177 radiotherapeutic counterpart.

## Materials and methods

### Peptides, radionuclides and chemicals

Na_2_EDTA, glycine, urea, NaOH, and citric acid were supplied from Sigma Aldrich (St. Louis, MO, USA), while ascorbic acid and phosphate buffer saline (PBS) tablets were purchased from Merck (Rahway, NJ, USA). The peptides AU-SAR-M1, AU-SAR-M2, and AU-SAR-M3, as well as the NOTA-PEG_2_-RM26 (NOTA = 1,4,7-triazacyclononane-1,4,7-triacetic acid) (Varasteh et al. 2013) were synthesised by Pepmic Co., Ltd. (Suzhou, Jiangsu, China), based on our design. The indium-111 chloride ([^111^In]InCl_3_) was supplied from Curium Pharma (Stockholm, Sweden).

### Radiolabeling: radiochemical studies

For labeling with In-111, an aliquot of test peptide solution (2 µL, 2 nmol, dissolved in MQ H_2_O) was mixed with NH_4_OAc buffer (60 µL, 0.2 M, pH 5.5), ascorbic acid (10 µL 0.1 M) and [^111^In]InCl_3_ solution (16 µL, 11–15 MBq). Activities were measured with a CRC-15R Dose Calibrator (Mirion technologies, Florham Park, NJ, USA). The reaction mixture was incubated for 30 min at 85 °C. Radiochemical yields were determined by instant thin layer chromatography (iTLC, strips from Agilent Technologies, Santa Clara, CA, USA) with 0.2 M citric acid as mobile phase and analysed using a phosphor TLC screen and Cyclone Plus imager (PerkinElmer®, Waltham, MA, USA).

Radiochemical purity was determined by reversed phase high performance liquid chromatography (RP-HPLC). The system was equipped with a LaPrep Sigma HPLC LP1100 pump (Hitachi High-Tech Corporation, Hitachinaka, Ibaraki, Japan) linked to a 40D LWL UV-detector with a 4 µL flow cell (Knauer, Berlin, Germany) and a Flow scan radioactivity detector (Bioscan, Paris, France) with an FC-3300 NaI/PMT radioactivity probe (Eckert & Ziegler, Berlin, Germany) and a manual sample injector 7725i (Rheodyne, Chrome Tech, Apple Valley, MN, USA,) fitted with a 20 µL sample loop (IDEX Health & Science, LLC, Rohnert Park, CA, USA). For data analysis and instrument monitoring the Open Lab EZChrome Elite software (Agilent, Santa Clara, CA, USA) was used. The column used was a Luna C18 column (5 µm, 100 Å, 150 × 4.6 mm from Phenomenex, Værløse, Denmark). For elution a two solvent gradient system was adopted, starting at 95% A/5% B up to 30% A/70% B at 15 min, going to 5% A/95% B at 17 min, remaining in this ratio for 2 min and returning to initial conditions over another 2 min (A: 0.1% trifluroacetic acid (TFA) in water; B: 0.1% TFA in acetonitrile, MeCN).

Radiometal-chelate stability was assessed by incubation of test radioligand (5 µL of the reaction mixture, 115 pmol) with a × 1000 molar excess of EDTA (115 nmol, 8.7 µL) for 1 h at room temperature. This result was compared to the same amount of radioligand left for 1 h at room temperature in an equivalent volume of PBS (8.7 µL). Samples were analysed by iTLC.

### Cell culture

The GRPR positive human prostate adenocarcinoma cell line PC-3 (Reile et al. 1994) was purchased from American Type Culture Collection (ATCC via LGC Standards AB, Borås, Sweden) and grown in Roswell Park Memorial Institute-1640 medium (RPMI-1640, Sigma Aldrich, Saint Louis, MO, USA) containing 1% (v/v) L-glutamine and supplemented with 10% (v/v) fetal bovine serum (FBS, Sigma Aldrich, St. Louis, MO, USA) as well as 1% (v/v) penicillin/streptomycin solution (PEST; 10 000 U/mL penicillin; 10 000 µg/mL streptomycin, Biochrom, Berlin, Germany). Cells were incubated in a 5% CO_2_ humidified atmosphere at 37 °C in 75 cm^2^ cell culture flasks with vent caps (Corning®, Corning, NY, USA) in a Sanyo MCO-19AIC incubator (SANYO Electric Co., Ltd, Osaka City, Osaka, Japan). For sub-culturing a 0.25% trypsin–EDTA solution (Sigma Aldrich, St. Louis, MO, USA) was used.

### In vitro* assays*

#### GRPR-specificity

For the determination of GRPR-specificity, PC-3 cells were seeded in 35 mm tissue-culture dishes (Corning®, Corning, NY, USA) and test radioligand was added to each well (1 nM). In a subset of dishes, 1 µM of the unlabeled NOTA-PEG_2_-RM26 was added for blocking the GRPR. Cells were incubated for 1 h at 37 °C and 5% CO_2_, washed with PBS, dissociated, collected, and sample-radioactivity was measured using a 2480 WIZARD^2^ automatic gamma counter (PerkinElmer®, Waltham, MA, USA). Experiments were performed in triplicate along with three standards for each compound.

#### GRPR binding affinity and kinetics

For evaluation of the receptor affinity and binding kinetics, PC-3 cells were seeded in 100 mm Nunc™ Petri dishes (Thermo Fisher, Waltham, MA, USA). A LigandTracer Yellow device (Ridgeview Instruments AB, Uppsala, Sweden) was used to measure the radiation from the Petri dish. Background with RPMI-1640 medium was measured for 30 min, after which 1 nM of radioligand was added and measured for 2 h. An additional concentration (3 nM) was measured for another 2 h, thereafter the medium was aspirated, replaced with fresh medium, and left running overnight to measure the dissociation rate. Data was fitted with the TracerDrawer software (Ridgeview Instruments AB, Uppsala, Sweden).

#### Cellular processing assay

PC-3 cells were seeded in 35 mm tissue-culture dishes. The next day, after aspiration of the medium and washing of the cells with PBS, 1 nM of test radioligand in complete growth medium was added to each dish and incubated at 37 °C in a 5% CO_2_ atmosphere for predetermined time points. At each timepoint the medium was aspirated, the dishes were washed with PBS and incubated with acid wash buffer (500 µL, 0.2 M glycine buffer, 0.15 M NaCl, 4 M Urea, pH 2) for 5 min on ice (membrane-bound fraction). Then the cells were washed with PBS and a basic solution (500 µL, 1 M NaOH) was added; the dishes were left to incubate at 37 °C in 5% CO_2_ for 30 min and scraped. Cell-lysates were collected (internalized fraction) and the activity of each fraction was measured on the gamma counter. The experiment was performed in triplicates.

### In vivo* experiments*

#### Metabolic stability

The metabolic stability of radioligands was tested by HPLC analysis of mice blood collected 5 min post-injection (pi). For the experiment, healthy Swiss Albino mice (30 ± 5 g body weight; > 8 weeks of age) were purchased from the NCSR “Demokritos” Animal House (Institute of Biosciences & Applications, NCSR “Demokritos”, Athens, Greece). Experiments were performed in accordance with the European guidelines in supervised and licensed facilities (EL 25 BIOexp 021) adhering to approved study protocols (Department of Agriculture and Veterinary Service of the Prefecture of Athens, protocol number #440,448, 01-06-2021).

Animals in control groups (*n* = 3) were injected intravenously (iv) with a bolus of test radioligand (100 μL, 2 nmol of peptide in PBS/EtOH v/v 9:1). Additional groups were pre-treated with a suspension of Entresto® (200 µL, 12 mg of Entresto®) by oral gavage 30 min prior to iv radioligand injection. The suspension was prepared from 200 mg pills (containing 24 mg sacubitril/26 mg valsartan, Novartis AG, Basel, Switzerland) turned to fine powder in a mortar, suspended in tap water and distributed in equal animal-individualized doses in Eppendorf tubes. Five min pi, the animals were euthanized and blood was collected directly from the heart in a prechilled heparinized syringe. Blood samples were immediately mixed with EDTA (0.1 mM, 20 μL) in LoBind Eppendorf tubes on ice. The mixture was centrifuged at 2000 g for 10 min at 4 °C and the plasma was collected and diluted with an equal volume of MeCN. After a second centrifugation at 15,000 g and 4 °C for 10 min, the supernatant was collected in a glass vial and concentrated under mild heating (50 °C) and a gentle stream on N_2_ (to 50–100 μL). Physiological saline was then added to the samples (final volume of 450–500 μL) and the resulting solutions were filtered through Millex GV filters (0.22 μm, 13 mm diameter, Millipore, Milford, CT, USA). To determine the percentage of intact peptide, aliquots were analysed by reversed phase radio-HPLC. For analyses a Symmetry Shield RP-18 (5 μm, 3.9 mm × 20 mm) cartridge column (Waters, Eschborn, Germany) was eluted with the following gradient: starting at 100% A–0% B and reaching to 60% A–40% B in 40 min (A: 0.1% v/v aqueous TFA, B: MeCN). A Waters Chromatograph connected to a Gabi gamma detector (Raytest RSM Analytische Instrumente GmbH, Straubenhardt, Germany) was used in analyses controlled by the Empower Software (Waters, Milford, MA, USA). The peak corresponding to the intact peptide was identified by co-injection of an aliquot of the labeling solution with the corresponding blood samples. Experiments were performed in triplicate and presented as average % intact radioligand ± standard deviation (sd).

#### Biodistribution and SPECT/CT imaging

Animal experiments were planned and performed in accordance with national legislation on protection of laboratory animals, and protocols were approved by the Ethics Committee for Animal Research in Uppsala, Sweden (5.8.18–00473/2021). BALB/c nu/nu female mice were xenografted by subcutaneous injection of PC-3 cells on the hind leg (7 × 10^6^ cells/mouse). At the date of the experiment, approximately 6 weeks after implantation, tumour sizes reached 0.11 ± 0.05 g. Groups of four mice were used for each time point. In the first group, mice were iv injected with test radiopeptide (30 kBq in 100 µL PBS supplemented with 1% bovine serum albumin (BSA), corresponding to 40 pmol) and euthanized at 4 h pi. Tumors and relevant organs were collected, weighted and sample radioactivity was measured using the gamma counter. In the case of [^111^In]In-AU-SAR-M1 two additional mice groups underwent the same procedure but were euthanized either at 24 h pi, or at 4 h pi following radioligand co-injection with excess (5 nmol) NOTA-PEG_2_-RM26 to determine the in vivo GRPR-specificity. For statistical analysis Two-Way Anova with Tuckey’s post hock analysis was employed, using GraphPad Prism 7 for Windows® (GraphPad Software, Boston, Massachusetts USA).

For SPECT/CT imaging, one mouse was injected with [^111^In]In-AU-SAR-M1 (1.2 MBq in 100 µL of 1% BSA in PBS, 80 pmol); for imaging the labeling was conducted as previously described but at a 10 MBq/nmol molar activity. Image acquisition was performed at 4 h pi under anaesthesia and at 24 h pi after euthanasia with CO_2_ asphyxiation. Whole body SPECT/CT scans were acquired using a NanoScan SPECT/CT instrument (Mediso Medical Imaging Systems, Budapest, Hungary). The acquisition time was 20 min. SPECT raw data were reconstructed using Tera-TomoTM 3D SPECT reconstruction technology (version 3.00.020.000; Mediso Medical Imaging Systems Ltd.). CT data were reconstructed using Filter Back Projection and fused with SPECT files with the Nucline 2.03 Software (Mediso Medical Imaging Systems Ltd.). The scans are presented as maximum intensity projections in the RGB colour scale.

## Results

### Radiolabeling: radiochemistry

**Fig. 2:**
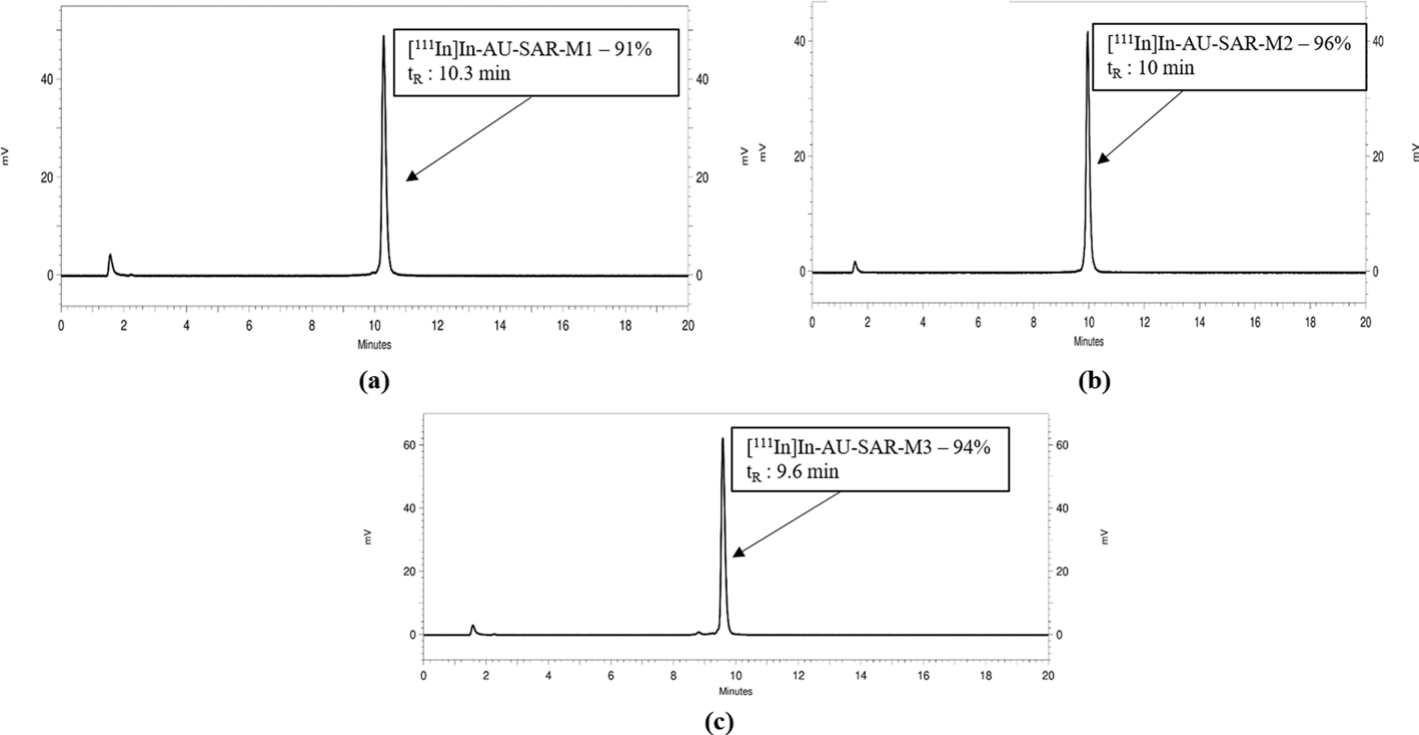
Representative HPLC radiochromatograms for **a** [^111^In]In-AU-SAR-M1—radiochemical purity: 91%, **b** [^111^In]In-AU-SAR-M2—radiochemical purity: 96%, (c) [^111^In]In-AU-SAR-M3—radiochemical purity: 94%

The three bioconjugates were successfully labeled with In-111 in an average radiochemical yield of > 97% (based on iTLC analysis of the reaction mixture) (Table 1). The identity of labeled peptides was confirmed using HPLC analysis (recovery ~ 90%), a single peak corresponding to non-labeled peptides was detected for all tested peptides (91.3 ± 0.8%, 96.3 ± 0.2% and 94.0 ± 0.4% for [^111^In]In-AU-SAR-M1, [^111^In]In-AU-SAR-M2 and [^111^In]In-AU-SAR-M3, respectively) (Fig. 2).

**Table 1:** Average labeling yield and in vitro stability tests in PBS and EDTA

[^111^In]In-AU-SAR-	M1	M2	M3
Average yield (%)	97.4 ± 2.6 (n = 7)	98.2 ± 1.8 (n = 5)	97.0 ± 3.0 (n = 6)
t_R_ (min)	10.3	10	9.6
PBS	95.3 ± 1.3%	99.0 ± 0.5%	95.1 ± 1.7%
EDTA	96.4 ± 0.5%	98.1 ± 0.1%	91.4 ± 0.7%
*p*-value (PBS-EDTA)	0.28	0.03	0.02

Data presented as net % sum area ± sd analysed by iTLC. Significance of difference between PBS and EDTA incubation presented with p-value

When assessing their radiometal-chelate stability, the radiolabeled compounds remained stable in the presence of a 1000-fold excess EDTA and PBS within 1 h at room temperature. None of the radioligands showed a significant In-111 release after 1 h incubation in PBS when compared by iTLC directly post labeling. By comparing the stability in PBS and EDTA, results for [^111^In]In-AU-SAR-M1 revealed no significant difference, while [^111^In]In-AU-SAR-M2 and [^111^In]In-AU-SAR-M3 showed a small, but significant difference in radiometal-chelate stability, which was more pronounced for [^111^In]In-AU-SAR-M3 (Table 1).

### In vitro* assays*

#### GRPR-specificity

**Fig. 3:**
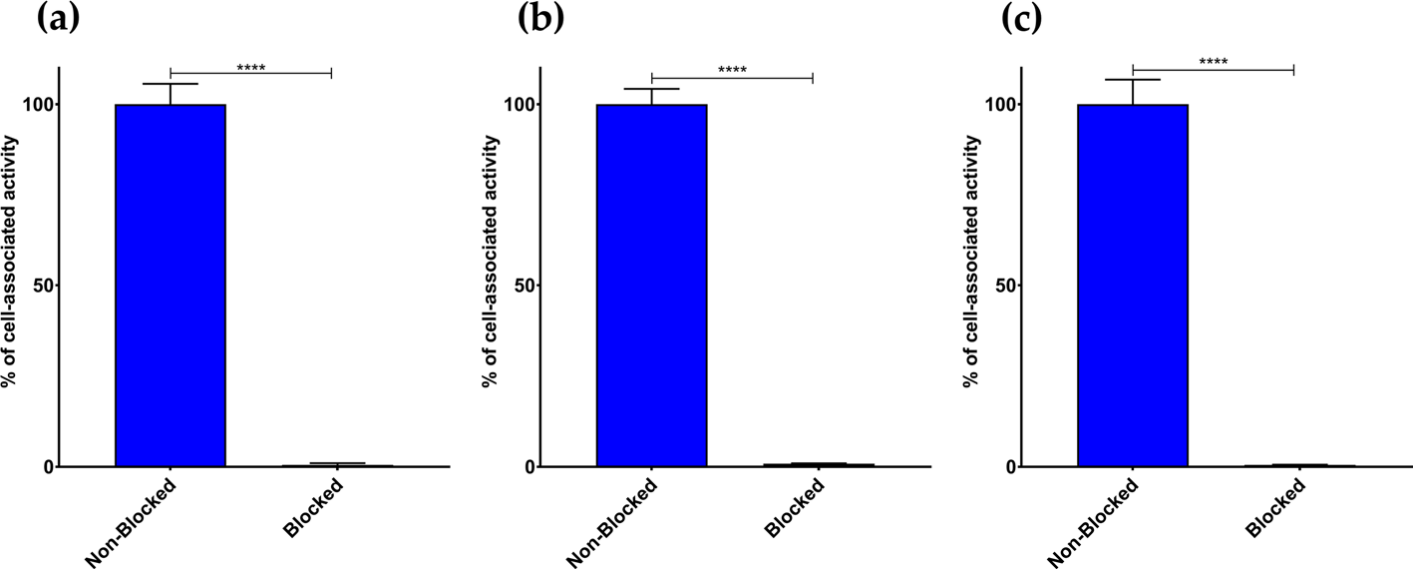
Percentage of activity linked to PC-3 cells in the absence (control—left blue bars) or in the presence of excess NOTA-PEG2-RM26 (blocked—right green, bars too low to be visible) for **a** [^111^In]In-AU-SAR-M1, **b** [^111^In]In-AU-SAR-M2 and **c** [^111^In]In-AU-SAR-M3 (**** = *p* < 0.0001)

The radioligands displayed high and GRPR-specific uptake in PC-3 cells during 1 h incubation at 37 °C. In the presence of an excess of NOTA-PEG2-RM26, to block the GRPR sites on the cells, these values dropped to < 1.5% for all three radioligands (*p* < 0.0001, Fig. 3), in agreement with a GRPR-mediated process.

#### GRPR-binding affinity and kinetics

**Fig. 4:**
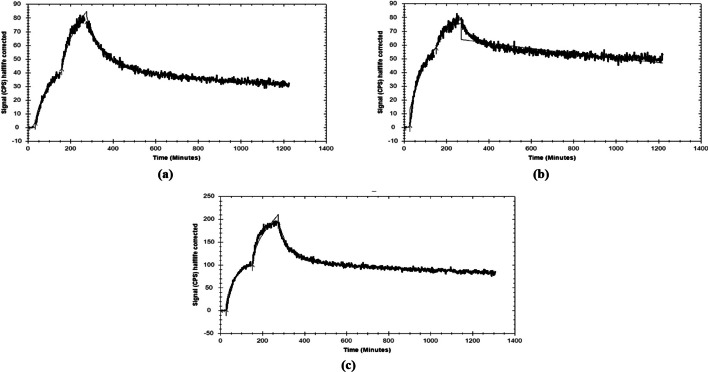
Ligandtracer sensograms for **a** [^111^In]In-AU-SAR-M1, **b** [^111^In]In-AU-SAR-M2 and **c** [^111^In]In-AU-SAR-M3; association was measured at 1 nM and 3 nM radioligand

The equilibrium dissociation constant K_D_ was determined by fitting the data to a 1:2 binding site model, which provided a superior fit over a 1:1 site model for [^111^In]In-AU-SAR-M1 and [^111^In]In-AU-SAR-M3. For [^111^In]In-AU-SAR-M2 the 1:1 site model gave a more reliable result (Fig. 4). As shown in Table 2, [^111^In]In-AU-SAR-M1 and [^111^In]In-AU-SAR-M3 showed similar affinity, in the sub-nanomolar to low nanomolar range, whilst [^111^In]In-AU-SAR-M2 had an approximately tenfold better affinity.

**Table 2:** Kinetics evaluation of the three compounds, performed with 1–3 nM radioligand

[^111^In]In-AU-SAR-	k_a_1(1/(M*s))	k_d_1 (1/s)	K_D_1 (nM)	k_a_2 (1/(M*s))	k_d_2 (1/s)	K_D_2 (nM)
M1	1.4 × 10^4^	5.4 × 10^–6^	0.39	1.9 × 10^5^	2.1 × 10^–4^	1.1
M2	1.8 × 10^5^	5.5 × 10^–6^	0.031			
M3	1.5 × 10^4^	4.4 × 10^–6^	0.30	3.1 × 10^5^	3.4 × 10^–4^	1.1

k_a_ = rate of association (1/(M*s)), k_d_ = rate of dissociation (1/s), K_D_ = equilibrium dissociation constant (nM)

#### Cellular processing assay

**Fig. 5:**
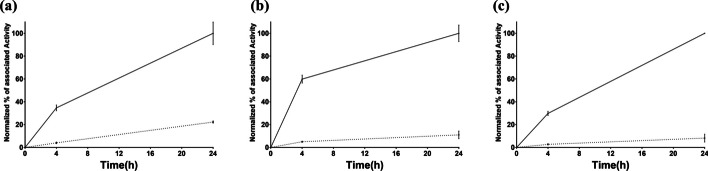
Normalized in vitro cellular processing showing % of total cell associated activity (solid line) and fraction of activity internalized (dotted line) for **a** [^111^In]In-AU-SAR-M1, **b** [^111^In]In-AU-SAR-M2 and **c** [^111^In]In-AU-SAR-M3

Cell-associated activity was studied at 4 and 24 h incubation in PC-3 cells at 37 °C; the percentage of internalized of the normalized cell-associated activity was 22.7 ± 1.0% for [^111^In]In-AU-SAR-M1, whilst lower for [^111^In]In-AU-SAR-M2 and [^111^In]In-AU-SAR-M3 at 24 h (11 ± 3% *p* < 0.01 and 8 ± 3%, *p* < 0.01, respectively) (Fig. 5).

### In vivo* experiments*

#### Metabolic study

As revealed by HPLC analysis of peripheral blood collected from healthy mice at 5 min pi, the three new radioligands showed similar metabolic stability with the [^99m^Tc]Tc-DB15 reference (Table 3). However, unlike [^99m^Tc]Tc-DB15, treatment of animals with Entresto® significantly increased their stability, indicating the involvement of NEP in their degradation (Table 3).

**Table 3:** In vivo stability comparing investigated compounds with [^99m^Tc]DB-15, without (controls) or during NEP-inhibition (induced by Entresto®, or phosphoramidon, PA)

Radioligand	Control	vs. ref	Between groups	+ Entresto®	vs. ref
[^99m^Tc]DB15	76 ± 2%		ns	83 ± 2% (PA)	
[^111^In]In-AU-SAR-M1	69 ± 2%	*	**	84 ± 7%	ns
[^111^In]In-AU-SAR-M2	72 ± 3%	ns	***	93.5 ± 0.8%	**
[^111^In]In-AU-SAR-M3	67 ± 3%	**	****	91 ± 1%	*

For statistical analysis, two-way ANOVA test was used, with Sidak post-hoc analysis for the multiple comparisons. ⁕: *p* < 0.05, ⁕⁕: *p* < 0.01, ⁕⁕⁕: *p* < 0.001, ⁕⁕⁕⁕: *p* < 0.0001, ns: non-significant

[^111^In]In-AU-SAR-M1 (*p* < 0.05) and [^111^In]In-AU-SAR-M3 (*p* < 0.01) showed a slightly but significantly lower stability compared to [^99m^Tc]DB15, whilst [^111^In]In-AU-SAR-M2 showed comparable stability. For the treated groups, [^111^In]In-AU-SAR-M1 showed an equivalent stability to [^99m^Tc]DB15, while the other two radioligands showed a higher stability compared to the reference compound (*p* < 0.01 and *p* < 0.05, respectively). Unlike [^99m^Tc]DB15 which was shown to be NEP-resistant, the new analogues showed a significant increase in stability between the control and the Entresto® treated groups, implying the involvement of NEP in their in vivo degradation.

#### Biodistribution and SPECT/CT imaging

**Fig. 6:**
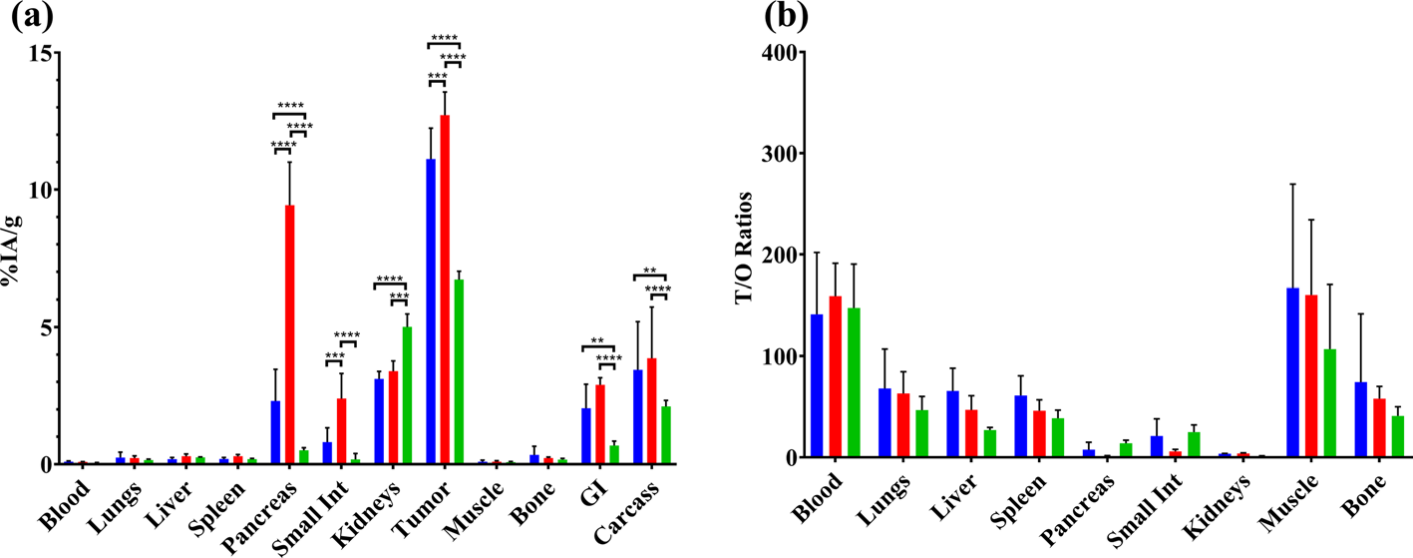
**a** Comparison of biodistribution results at 4 h pi across [^111^In]In-AU-SAR-M1 (blue bars), [^111^In]In-AU-SAR-M2 (red bars) and [^111^In]In-AU-SAR-M3 (green bars) (from left to right), presented as average %IA/g ± sd (error bars), n = 4. Results for gastrointestinal tract (GI) and carcass are presented as average %IA ± sd.** = *p* < 0.01; *** = *p* < 0.001; and **** = *p* < 0.0001; **b** T/O ratios for across [^111^In]In-AU-SAR-M1 (blue bars), [^111^In]In-AU-SAR-M2 (red bars) and [^111^In]In-AU-SAR-M3 (green bars) at 4 h pi

The biodistribution results for [^111^In]In-AU-SAR-M1, [^111^In]In-AU-SAR-M2 and [^111^In]In-AU-SAR-M3 are summarized in Fig. 6a and Additional file 1: Table S1, while the tumor-to-organ ratios (T/O) are given in Fig. 6b and Additional file 1: Table S2. Results are presented as average %IA/g ± sd, except for gastrointestinal tract (GI) and carcass, which are presented as average %IA ± sd.

**Fig. 7:**
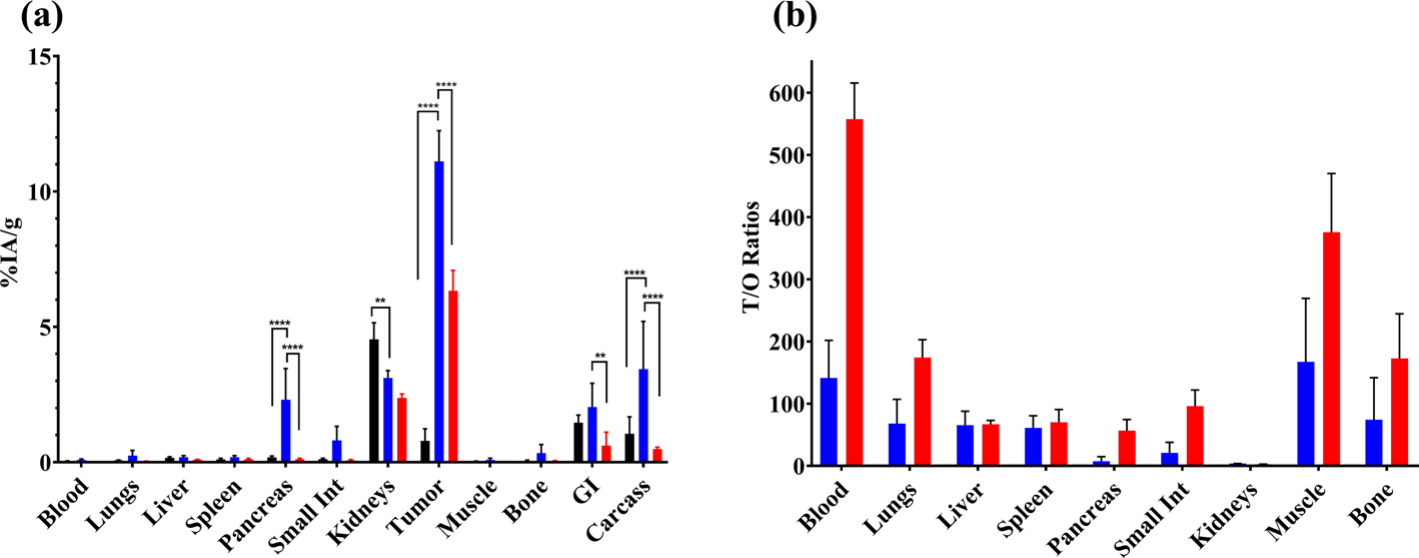
**a** Biodistribution results of (from left to right): GRPR-specificity (black bars), [^111^In]In-Au-SAR-M1 at 4 h (blue bars) and 24 h (red bars). Results are given as average %IA/g ± sd (error bars), ** = *p* < 0.01; **** = *p* < 0.0001. **b** T/O ratios for [^111^In]In-AU-SAR-M1 at 4 h pi (blue bars) and 24 h pi (red bars), given as mean values ± sd (as error bars), n = 4 for both groups

The biodistribution profiles of [^111^In]In-AU-SAR-M1 and [^111^In]In-AU-SAR-M2 were similar, while [^111^In]In-AU-SAR-M3 showed more pronounced differences. Thus, the tumor uptake led to the following rank of analogues: [^111^In]In-AU-SAR-M2 > [^111^In]In-AU-SAR-M1 (*p* < 0.001) >  > [^111^In]In-AU-SAR-M3 (*p* < 0.0001 compared with the previous two). On the other hand, the highest kidney uptake was displayed by [^111^In]In-AU-SAR-M3 (*p* < 0.05). Uptake in the GRPR-rich mouse pancreas was markedly higher for [^111^In]In-AU-SAR-M2 (> fourfold higher than [^111^In]In-AU-SAR-M1 and > 18-fold higher than [^111^In]In-AU-SAR-M3; *p* < 0.0001 in both cases) and small intestines (*p* < 0.001 & *p* < 0.0001, respectively). With regards to liver uptake, [^111^In]In-AU-SAR-M1 showed the lowest, but not significantly different, values compared with the other compounds. When T/O ratios are considered, [^111^In]In-AU-SAR-M1 showed better (but not significantly higher) ratios for lungs, liver, muscle, bone and a lower ratio for pancreas in comparison with [^111^In]In-AU-SAR-M3. In light of these results, [^111^In]In-AU-SAR-M1 was chosen for further studies to determine in vivo GRPR-specificity of uptake at 4 h pi as well as to assess its biodistribution at 24 h pi (Fig. 7, Additional file 1: Table S1).

As shown in Fig. 7a, a significant decrease in pancreatic uptake (2 ± 1%IA/g controls vs. 0.18 ± 0.05%IA/g in block, *p* < 0.0001) as well as in tumor uptake (11 ± 1%IA/g controls vs. 0.8 ± 0.4%IA/g in block, *p* < 0.0001) was observed between the animals coinjected with excess of the GRPR-antagonist NOTA-PEG_2_-RM26 compared to controls, further implying a GRPR-mediated process. Kidney uptake was significantly higher in the blocked animals (3.1 ± 0.3%IA/g controls vs. 4.5 ± 0.6%IA/g in block, *p* < 0.01). By comparing the 4 h and 24 h biodistribution of [^111^In]In-AU-SAR-M1, we firstly observe a rapid clearance from the GRPR-rich mouse pancreas with only 5% of initial uptake remaining at 24 h pi in this tissue. On the other hand, the tumor uptake decreased approximately by 40% within the same time frame (11 ± 1%IA/g at 4 h pi to 6.3 ± 0.7%IA/g at 24 h pi, *p* < 0.0001). The kidney uptake remained on the same level with a tendency to decrease over time (*p* > 0.05).

An increasing T/O ratio over time was established for almost all organs (Fig. 7b) with the exception of the kidneys. Tumor-to-blood showed a > threefold increase over time (from 160 at 4 h pi to > 550 at 24 h pi). The tumor-to-liver ratio remained approximately the same, while the tumor-to-muscle ratio increased by about 1.8-fold. The tumor-to-bone ratio remained high (> 150) and increased only marginally at 24 h pi. The tumor-to-pancreas ratio showed an excellent increase, approximately sixfold at 24 h pi (9 ± 11 to 57 ± 18), as consistent with a radioantagonist profile (Abouzayed et al. 2023; Damiana et al. 2023).

**Fig. 8:**
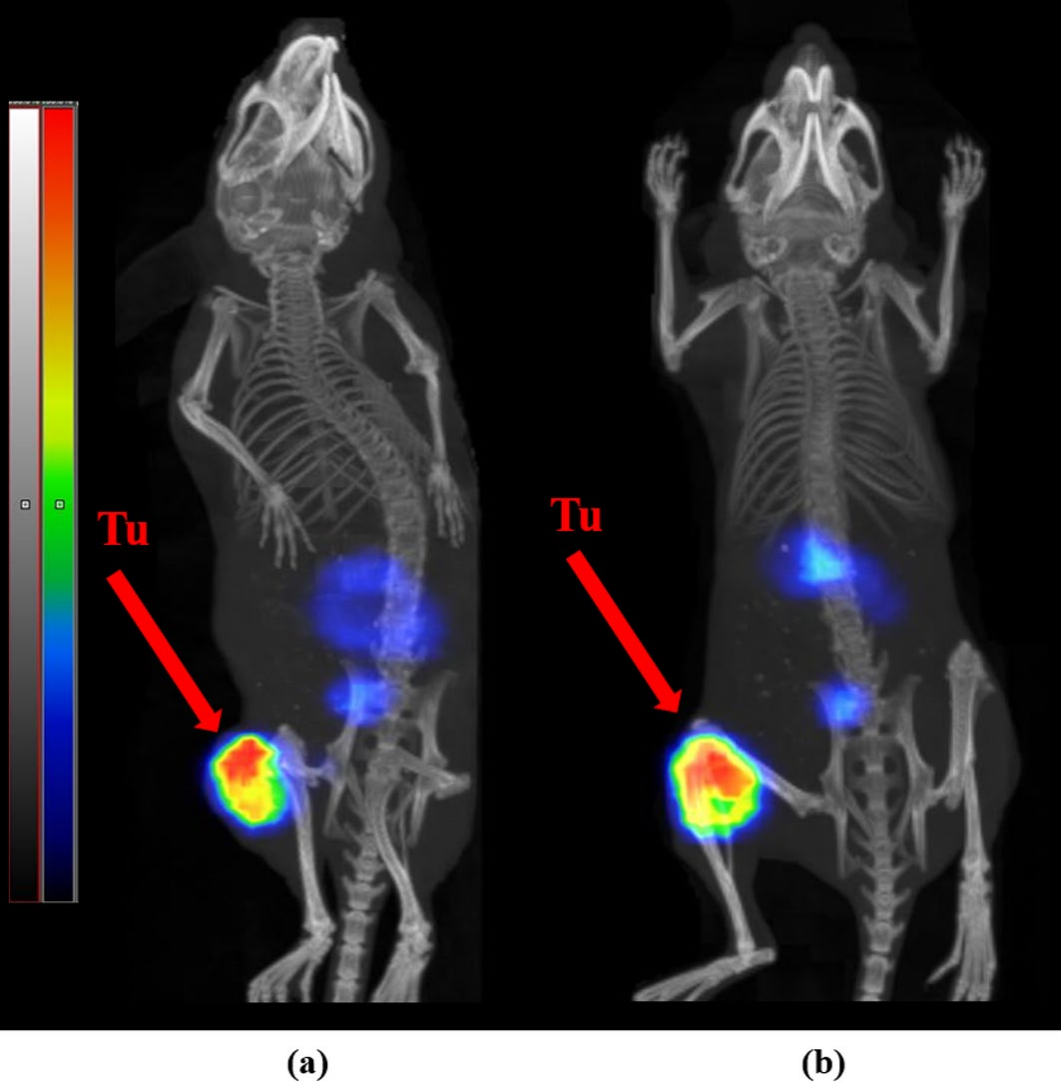
SPECT/CT images of a PC-3 tumor-bearing mouse, at **a** 4 h and **b** 24 h pi of [^111^In]In-AU-SAR-M1, red arrows indicate tumor-sites

SPECT/CT imaging of [^111^In]In-AU-SAR-M1 was conducted at 4 h and 24 h pi and representative images are included in Fig. 8. Tumors were well visualized in both time-points along with the kidneys against a very clear background, in agreement with biodistribution results.

## Discussion

Targeting of GRPR by means of peptide radionuclide-carriers has been regarded as a promising approach in the treatment of prostate cancer (Abouzayed et al. 2020; Dumont et al. 2013; Kurth et al. 2020; Mitran et al. 2019). Based on the GRPR overexpression in the early stages of the disease as opposed to healthy or hyperplastic surrounding tissue, GRPR-directed radiopharmaceuticals are expected to play a significant role in the management of prostate cancer, especially in primary and oligometastatic stage (Beer et al. 2012; Mansi et al. 2021; Patel et al. 2006; Rinne et al. 2021). The first-in-human study employing the therapeutic [^177^Lu]Lu-RM2 radioligand to assess this approach, confirmed the excellent tolerability of the GRPR-antagonist. Moreover, over-expression of GRPR was reported in other cancers, among the most relevant for use of a GRPR targeting radiotherapy are gastrinomas (about 100% express GRPR), gastrointestinal stromal tumors (GIST, about 85%) and estrogen receptor positive breast cancer (over 80%) (Cornelio et al. 2007; Dimitrakopoulou-Strauss et al. 2007; Morgat et al. 2017; Reubi et al. 2004; Reubi et al. 2002). Clinical data demonstrates high uptake of GRPR-targeting antagonistic peptides in patients with estrogen receptor positive breast cancer both in primary lesions, lymph node and bone metastases (Chernov et al. 2023; Nock et al. 2021; Zang et al. 2018).

Yet, two major problems of GRPR-antagonist radioligands need to be resolved to maximise efficacy, to reduce pancreatic uptake and to enhance metabolic stability in circulation (Kurth et al. 2020).

Amongst the radiolabeled GRPR-antagonists developed in recent years (Mansi et al. 2021; Nock et al. 2023), [^99m^Tc]Tc-DB15 has especially attracted our attention by combining a high GRPR-affinity, resistance to NEP in the bloodstream, good tumor uptake and excellent tolerability in patients (Nock et al. 2021). In an attempt to reproduce these promising qualities in theranostics beyond SPECT/CT diagnostic imaging alone, trivalent radiometals were inevitably considered, and especially the In-111/Lu-177 theranostic pair. Toward this goal, the N-terminal N_4_ of [^99m^Tc]Tc-DB15 was replaced by the DOTAGA-chelator in the first antagonist AU-SAR-M1 presented herein, inadvertently leading to the loss of a positive charge attributed to the [^99m^Tc]Tc(O)_2_(N_4_)]^+1^ radiometal-chelate (Nock et al. 2021). To counterbalance this loss, potentially impairing GRPR-affinity (Zhang et al. 2007), a basic _L_Arg or _D_Arg residue was introduced next to DOTAGA-chelator in AU-SAR-M2 and AU-SAR-M3, respectively. The impact of the above structural modifications on the respective [^111^In]In-radioligands was assessed in a series of in vitro and animal assays, based on GRPR-expressing human cancer cells, to reveal the most promising candidate for further evaluation after labeling with Lu-177. Eventually, a well-characterized radiotheranostic pair for translation in prostate cancer patients could become available directly or via the new information acquired in this work.

After successful labeling with In-111, the three radioligands were tested in PC-3 cells to evaluate their cell uptake pattern and their GRPR-specificity. As it shown in Fig. 3, the radiotracers displayed high cellular association, which was clearly GRPR-driven. Furthermore, the conjugates retained a typical radioantagonist profile with the bulk of the cell-associated activity remaining bound on the cell membrane and slowly internalizing over time (Fig. 4).

The GRPR-affinity studies revealed a few interesting results (Table 2). First, [^111^In]In-AU-SAR-M2 exhibited an order of magnitude higher receptor affinity than the other two analogues, a finding attributed to the positive charge of L-Arg next to DOTAGA (Zhang et al. 2007). Second, the real time measured affinities for [^111^In]In-AU-SAR-M1 and [^111^In]In-AU-SAR-M3 were found similar to the affinity of [^111^In]In-DOTAGA-PEG_2_-RM26 (K_D_ = 0.44 ± 0.05 nM) and its theranostic counterpart [^177^Lu]Lu-DOTAGA-PEG_2_-RM26 (K_D_ = 0.4 ± 0.2 nM) (Mitran et al. 2019; Mitran et al. 2016). Third, [^111^In]In-AU-SAR-M2 and [^111^In]In-AU-SAR-M3 displayed different dissociation constants, despite the presence of the positive charge next to DOTAGA in both due to the basic _L/D_Arg. This unexpected finding implies that the different 3D-configuration at the N-terminal of the peptide (_L_Arg vs. _D_Arg) interferes with the interaction of the whole molecule with the GRPR. In the case of _D_Arg, the impact of such disadvantageous molecule configuration turned out to overwhelm the influence of the positive charge introduced by the basic residue (Table 2). Furthermore, the fact that [^111^In]In-AU-SAR-M2 demonstrated only 1:1 interaction with GRPR, unlike [^111^In]In-AU-SAR-M3 displaying 1:2 interaction (with very close rates of association and dissociation), corroborates this hypothesis. In fact, affinity data for [^111^In]In-AU-SAR-M2, were analysed by a 1:2 model as well, but the K_D__1_ found in the range of 10^-20^ M turned out to be unrealistic for accurate measurements, leaving the 1:1 model as the only rational option.

According to the metabolic stability results in mice peripheral blood, [^111^In]In-AU-SAR-M2 was as stable as [^99m^Tc]Tc-DB15, with [^111^In]In-AU-SAR-M1 and [^111^In]In-AU-SAR-M3 showing somewhat lower stability (Table 3). Unlike [^99m^Tc]Tc-DB15 previously shown to be NEP-resistant (Nock et al. 2021), the new radioligands showed significant stability increase in the blood of mice treated with Entresto® in vivo releasing the potent and selective NEP inhibitor sacubitrilat. This finding implicates NEP in their in vivo degradation (Kanellopoulos et al. 2020). It appears that exchange of the radiometal-chelate from [[^99m^Tc]Tc(O)_2_(N_4_)]^+1^ in [^99m^Tc]Tc-DB15 to [[^111^In]In(DOTAGA)]^−1^ in [^111^In]In-AU-SAR-M1 rendered [^111^In]In-AU-SAR-M1 more vulnerable to the fast proteolytic action of the peptidase. Furthermore, introduction of _L_Arg next to DOTAGA in [^111^In]In-AU-SAR-M2 improved its stability against NEP, whereas the presence of _D_Arg in [^111^In]In-AU-SAR-M3 resulted in lower stability. The observed differences may represent distinct conformations of each molecule, leading to discrete ways of accessing the active centre of the enzyme, and their elucidation requires further dedicated studies. The different resistance of the new radioligands to NEP, as well as the degree of their stabilization by Entresto® should be taken into account in view of treatment options. Radioligand stabilization applying a fully characterized registered drug, like Entresto®_,_ is expected to enhance their tumor-targeting capabilities, a highly desirable feature for targeted radiotherapy with a therapeutic radionuclide such as Lu-177 (Kanellopoulos et al. 2020; Nock and Maina 2019).

As expected, the higher GRPR-affinity and better metabolic stability of [^111^In]In-AU-SAR-M2 translated in higher tumor uptake in PC-3 xenograft-bearing mice. On the other hand, [^111^In]In-AU-SAR-M3 demonstrated the lowest tumor uptake (Additional file 1: Table S1), although its GRPR-affinity and in vivo stability were comparable with [^111^In]In-AU-SAR-M1 (Fig. 3, Table 2, 3). Furthermore, [^111^In]In-AU-SAR-M3 displayed the highest kidney uptake, but the lowest blood values and whole body retention of activity (Additional file 1: Table S1). These findings, taken together, indicate that receptor-affinity and metabolic stability, while crucial, are not the exclusive factors dominating the biodistribution profile. Among the three radioligands, [^111^In]In-AU-SAR-M2 showed the highest uptake in small intestines and pancreas, both GRPR-rich organs (Körner et al. 2014; Reubi 2003), most probably as a result of its higher GRPR-affinity in comparison with the other two compounds. The tumor and pancreatic uptake were shown to be GRPR-specific during an additional GRPR-blockade study in the case of [^111^In]In-AU-SAR-M1 (Fig. 7a). Overall, more favourable T/O ratios were achieved by [^111^In]In-AU-SAR-M1 amongst the three radioligands and hence its biodistribution was additionally assessed at 24 h pi. The background activity declined from 4 to 24 h pi, including the GRPR-rich mouse pancreas and small intestines, but the kidney uptake remained at the same level. Tumor uptake dropped to 57% of the 4 h pi value. However, as the decrease of activity levels in other organs/tissues was faster (with the exception of the kidneys), T/O ratios increased, as typically observed for receptor antagonists (Lymperis et al. 2019).

Based on the above, [^111^In]In-AU-SAR-M1 turned out to be a new promising diagnostic tool for diagnosis using SPECT/CT, combining a high tumor uptake with a very clear background at 4 h pi already. Furthermore, due to its good tumor retention and faster background clearance, [^111^In]In-AU-SAR-M1 was able to provide excellent images at both 4 and 24 h pi, indicating the promising translation prospects to the respective therapeutic [^177^Lu]Lu-AU-SAR-M1 counterpart.

The tumor-to-kidney ratios for [^111^In]In-AU-SAR-M1 were 3.6 ± 0.2 at 4 h pi and 2.7 ± 0.2 at 24 h pi. These values are comparable with the data for the [^177^Lu]Lu-DOTAGA-PEG2-RM26 (2.8 ± 0.6 at 1 h pi and 3.1 ± 0.3 at 24 h pi) that in preclinical level demonstrated its utility to GRPR targeting therapy without development of nephrotoxicity (Mitran et al. 2019). The increase of tumor-to-kidney absorbed dose ratio might improve therapeutic potential of [^177^Lu]Lu-AU-SAR-M1. Three strategies are proposed in literature for such improvement: i. structural modification of peptide-conjugate, ii. chemically-induced reduction of renal uptake, and iii. drug-induced reduction of radiation damage to the kidney (Geenen et al. 2021). Structural modification of small-size peptides may induce drastic changes in their biodistribution profile, as demonstrated in the present study. Moreover, interventions leading to overall charge changes of the peptide-radioligand may negatively affect GRPR-binding affinity. Alteration of biological properties may be caused as well by simple switch of radiometal used for labeling (Lymperis et al. 2018). It should be noted that such a radiometal switch, is the next phase in our studies, namely the exchange of the diagnostic SPECT radionuclide In-111 to its radiotherapeutic counterpart Lu-177. According to previous experience, the transition from In-111 to Lu-177 may lead to comparable or even superior biodistribution profile (Mitran et al. 2019; Mitran et al. 2016). Further pharmacokinetic improvements may occur during in situ NEP-inhibition, as previously reported for [^177^Lu]Lu-DOTAGA-PEG2-RM26 (Mitran et al. 2019). In animals treated with the potent NEP-inhibitor phosphoramidon, the tumor-to-kidneys ratio increased to 3.8 ± 0.9 from 2.7 ± 0.4 in control group due to an increase in tumor uptake. We herein have shown that NEP-inhibition could induced using a fully characterized, commercially available registered drug (e.g. Entresto®) (Gu et al. 2010; McMurray et al. 2014)), facilitating clinical translation. Further studies to validate these prospects in animal models are currently on the way aiming for eventual proof-of-principle assessments in prostate cancer patients. Interestingly, several interventions for reduction of renal uptake, such as lysine/arginine or gelofusine infusion, as well as careful adjustment of the optimal injected peptide dose/amount have been successfully used in the clinic (Geenen 2021).

## Conclusions

Among the three new [^99m^Tc]Tc-DB15-base analogues designed to accommodate trivalent radiometals for theranostic use, [^111^In]In-AU-SAR-M1 turned out to be the most promising, owing to its high GRPR-specific tumor uptake and retention in combination with a low and declining uptake in healthy tissues. The in situ stabilization of the radioligand in the blood-stream using Entresto® (as a source of the potent and selective NEP-inhibitor sacubitrilat), is expected to further upgrade the biodistribution profile of [^111^In]In-AU-SAR-M1 and possibly its theranostic partner [^177^Lu]Lu-AU-SAR-M1 as well. The renal uptake of [^111^In]In-AU-SAR-M1 in absence of treatment with NEP inhibitor remained 2.5-fold lower than the tumor uptake at 24 h pi. This may still be the limiting factor for therapeutic applications in patients, potentially requiring NEP-inhibition and kidney protection regimens. Further studies are warranted to establish the radiotheranostic value of the [^111^In]In-AU-SAR-M1/[^177^Lu]Lu-AU-SAR-M1 pair, alone or during NEP-inhibition, in the management of prostate cancer or other GRPR-expressing malignancies.

### Supplementary Information

**Additional file 1. Table S1.** Biodistribution results. **Table S2.** Average T/O ratios.

## Data Availability

All data generated or analyzed during this study are included in this published article and its supplementary information files.

## Abbreviations

DOTAGA: 1,4,7,10-Tetraazacyclododecane-1-glutaric acid-4,7,10-triacetic acid
EDTA: Ethylenediaminetetraacetic acid
GI: Gastrointestinal tract
GRPR: Gastrin-releasing peptide receptor
iv: Intravenous
NEP: Neprilysin
PBS: Phosphate buffer saline
PC: Prostate cancer
PET: Positron emission tomography
pi: Post injection
PSMA: Prostate specific membrane antigen
SPECT: Single photon emission computed tomography
T/O: Tumor-to-organ ratios
%IA/g: % Injected activity per gram
