# Supplementary material for: Preclinical evaluation of new GRPR-antagonists with improved metabolic stability for radiotheranostic use in oncology
Source: EJNMMI Radiopharm Chem. 2024 Feb 16;9:13. doi: 10.1186/s41181-024-00242-6 (PMC10873254; doi:10.1186/s41181-024-00242-6)
Supplement: Supplementary file 1 — Additional file 1. Table S1. Biodistribution results. Table S2. Average T/O ratios. [file 41181_2024_242_MOESM1_ESM.docx]

# Supplementary

***Table S1.*** *Biodistribution results as average %IA/g ± sd (n =4), for the three [^111^In]In-AU-SAR-Mx, x= 1, 2 or 3. For GI (gastrointestinal tract) and carcass uptake values are given as %IA.*

| Organ | M1 | | | M2 | M3 |
| --- | --- | --- | --- | --- | --- |
|  | **4 h block** | **4 h pi** | **24 h pi** | **4 h pi** | **4 h pi** |
| Blood | 0.04 ± 0.01 | 0.08 ± 0.03 | 0.01 ± 0.001 | 0.08 ± 0.01 | 0.05 ± 0.01 |
| Lungs | 0.07 ± 0.01 | 0.2 ± 0.2 | 0.04 ± 0.003 | 0.2 ± 0.1 | 0.15 ± 0.03 |
| Liver | 0.16 ± 0.04 | 0.2 ± 0.1 | 0.09 ± 0.01 | 0.3 ± 0.1 | 0.25 ± 0.02 |
| Spleen | 0.10 ± 0.04 | 0.12 ± 0.05 | 0.10 ± 0.04 | 0.3 ± 0.1 | 0.18 ± 0.03 |
| Pancreas | 0.18 ± 0.05*^a^* | 2 ± 1*^a,b,c,d^* | 0.12 ± 0.03*^b^* | 9 ± 2*^c,e^* | 0.5 ± 0.1*^d,e^* |
| Small intestines | 0.11 ± 0.04 | 0.8 ± 0.5*^c^* | 0.07 ± 0.03 | 2.4 ± 0.9*^c,e^* | 0.2 ± 0.2*^e^* |
| Kidneys | 4.5 ± 0.6*^a^* | 3.1 ± 0.3*^a,d^* | 2.38 ± 0.14 | 3.4 ± 0.4*^e^* | 5.0 ± 0.5*^d,e^* |
| Tumor | 0.8 ± 0.4*^a^* | 11 ± 1*^a,b,c,d^* | 6.3 ± 0.8*^b^* | 12.7 ± 0.8*^c,e^* | 6.7 ± 0.3*^d,e^* |
| Muscle | 0.03 ± 0.01 | 0.09 ± 0.06 | 0.02 ± 0.01 | 0.09 ± 0.04 | 0.07 ± 0.03 |
| Bone | 0.06 ± 0.02 | 0.3 ± 0.3 | 0.04 ± 0.02 | 0.23 ± 0.04 | 0.17 ± 0.04 |
| GI | 1.5 ± 0.3 | 2.0 ± 0.9*^b,d^* | 0.62 ± 0.49*^b^* | 2.9 ± 0.3*^e^* | 0.7 ± 0.2*^d,e^* |
| Carcass | 1.1 ± 0.6*^a^* | 4 ± 2*^a,b^* | 0.48 ± 0.07*^b,d^* | 4 ± 2*^e^* | 2.1 ± 0.2*^d,e^* |

*a: statistical difference between 4 h block and 4 h pi for [^111^In]In-AU-SAR-M1*

*b: statistical difference between 4 h and 24 h pi for [^111^In]In-AU-SAR-M1*

*c: statistical difference between [^111^In]In-AU-SAR-M1 and [^111^In]In-AU-SAR-M2 at 4 h pi*

*d: statistical difference between [^111^In]In-AU-SAR-M1 and [^111^In]In-AU-SAR-M3 at 4 h pi*

*e: statistical difference between [^111^In]In-AU-SAR-M2 and [^111^In]In-AU-SAR-M3 at 4 h pi*

***Table S2.*** *Average T/O ratios ± sd (n=4) for the three [^111^In]In-AU-SAR-Mx, x= 1, 2 or 3.*

| Organ | M1 | | M2 | M3 |
| --- | --- | --- | --- | --- |
|  | **4 h pi** | **24 h pi** | **4 h pi** | **4 h pi** |
| Blood | 141 ± 61*^a^* | 557 ± 58 *^a^* | 159 ± 32 | 147 ± 43 |
| Lungs | 68 ± 39 *^a^* | 174 ± 29 *^a^* | 63 ± 22 | 47 ± 14 |
| Liver | 65 ± 22 | 67 ± 6 | 47 ± 14 | 27 ± 3 |
| Spleen | 61 ± 19 | 70 ± 20 | 46 ± 11 | 39 ± 8 |
| Pancreas | 8 ± 7 | 57 ± 18 | 1.4 ± 0.3 | 14 ± 3 |
| Small intestines | 21 ± 17 *^a^* | 96 ± 26 *^a^* | 6 ± 2 | 25 ± 7 |
| Kidneys | 3.6 ± 0.2 | 2.7 ± 0.2 | 3.8 ± 0.6 | 1.4 ± 0.1 |
| Muscle | 167 ± 102 *^a^* | 375 ± 95 *^a^* | 161 ± 74 | 107 ± 63 |
| Bone | 74 ± 68 *^a^* | 173 ± 72 *^a^* | 58 ± 12 | 41 ± 9 |

*a: statistical difference between 4 h pi and 24 h pi for [^111^In]In-AU-SAR-M1*
